# Synthesis and stereochemical determination of an antiparasitic *pseudo*-aminal type monoterpene indole alkaloid

**DOI:** 10.1007/s11418-016-1012-2

**Published:** 2016-06-21

**Authors:** Yoshihiko Noguchi, Tomoyasu Hirose, Aki Ishiyama, Masato Iwatsuki, Kazuhiko Otoguro, Toshiaki Sunazuka, Satoshi Ōmura

**Affiliations:** Kitasato Institute for Life Sciences, Kitasato University, 5-9-1 Shirokane, Minato-ku, Tokyo, 108-8641 Japan; Graduate School of Infection Control Sciences, Kitasato University, 5-9-1 Shirokane, Minato-ku, Tokyo, 108-8641 Japan

**Keywords:** 5-Nor stemmadenine alkaloid, Antimalarial agent, *Pseudo*-aminal type structure, Iminophosphorane mediated cascade reaction, Chirality transfer intramolecular Michael reaction, Diastereoselective 1,2-addition using indole nucleophile

## Abstract

5-Nor stemmadenine alkaloids, isolated from the genus *Tabernaemontana*, display a range of bioactivity. 16-Hydroxy-16,22-dihydroapparicine, the active component of an extract from the *Tabernaemontana* sp. (*dichotoma*, *elegans*, and *divaricate*), exhibited potent antimalarial activity, representing the first such report of the antimalarial property of 5-nor stemmadenine alkaloids. We, therefore, decided to attempt the total synthesis of the compound to explore its antimalarial activity and investigate structure and bioactivity relationships. As a result, we completed the first total synthesis of 16-hydroxy-16,22-dihydroapparicine, by combining a phosphine-mediated cascade reaction, diastereoselective nucleophilic addition of 2-acylindole or methylketone via a Felkin–Anh transition state, and chirality transferring intramolecular Michael addition. We also clarified the absolute stereochemistries of the compound. Furthermore, we evaluated the activity of the synthetic compound, as well as that of some intermediates, all of which showed weak activity against chloroquine-resistant *Plasmodium falciparum* (K1 strain) malaria parasites.

## Introduction

Naturally occurring chemicals represent a treasure trove of compounds which hold promise as the seeds of discovery for drugs and medicines and which may facilitate the elucidation of structure and function investigations of bioactivity [1]. Ōmura’s research group at the Kitasato Institute is a global pioneer in the search for bioactive agents that may be of use in developing drugs and medicines to fight to infection and combat tropical diseases (such as the filariases, malaria, trypanosomiasis, etc.), all originating from microbial metabolites. At present, 483 new compounds have been discovered, 26 of which have become useful, widely used agents in human and animal health, including the ground-breaking avermectins [2].

**Fig. 1:**
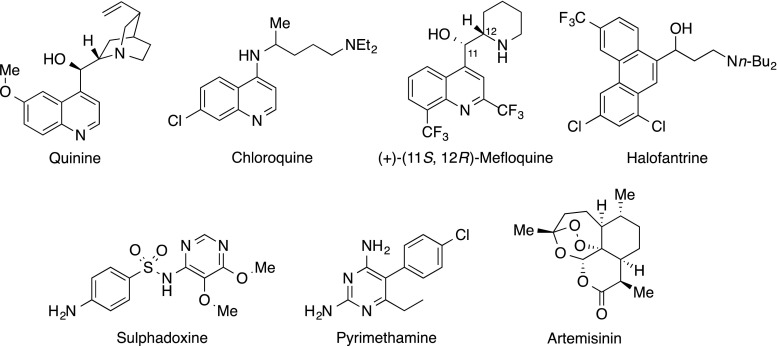
Therapeutic drugs for malaria

Malaria is one of the world’s worst health and socioeconomic problems, causing widespread death, disease, disability, and economic loss. Infection arises when a protozoal parasite of the *Plasmodium* genus is transmitted to humans via the bites of blood-feeding mosquitoes. *Plasmodium falciparum* parasites cause the most deadly form of the disease, which can cause death in a few days, especially if cerebral malaria develops. Generally, most deaths occur in children under 5 years old, although deaths have been reduced markedly by recent global initiatives to tackle the disease [3–5]. Commonly used drugs to combat malaria include quinine, chloroquine, mefloquine, halofantrine, and sulfadoxine/pyrimethamine (Fig. 1). However, drug resistance in parasites has usually developed quickly, rendering many of these drugs useless, preventing effective treatment and hindering disease elimination efforts. In 1972, Professor Tu Youyou discovered artemisinin to be the active ingredient in the plant *Artemisia annua*, which was commonly used in China to treat fever. Artemisinin derivatives became the most effective therapeutic drugs against malaria [6]. The World Health Organization (WHO) recommends artemisinin-based combination therapies (ACTs) for malaria treatment [7], a multidrug approach requiring the use of artemisinin together with other drugs to help offset the pace of drug resistance to artemisinin developing and spreading. ACTs are already compromised because the safety of artemisinin with regard to use during first trimester pregnancy is yet to be established and, worse, resistance to artemisinin derivatives developed almost immediately in locations along the Thai–Cambodian border [8–11]. Therefore, inexpensive and potent antimalarial drugs, especially those that have different modes of action, are urgently required on a probably continuing basis due to the ability of the malaria parasites to quickly develop drug resistance.

Many of the therapies currently in development use known antimalarial pharmacophores (e.g., aminoquinolines and/or peroxides), which have been chemically modified to overcome the failures of their predecessors [12]. Although these compounds have been important in the treatment of malaria, it would be highly advantageous to discover chemotypes with novel action mechanisms [13]. However, despite important advances in our understanding of the *Plasmodium* genome, the identification and validation of new drug targets have been challenging [14–16].

**Fig. 2:**
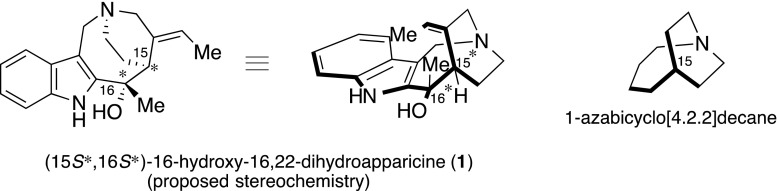
Structure of (15*S*,16*S*)-16-hydroxy-16,22-dihydroapparicine (**1**)

16-Hydroxy-16,22-dihydroapparicine (**1**), a known 5-nor stemmadenine alkaloid, was identified at the Kitasato Institute as a main component of a leaf’s MeOH extract from the plant *Tabernaemontana dichotoma*, which displayed antimalarial properties. The potent antimalarial activity of the complex leaf extract against chloroquine-resistant *Plasmodium falciparum* (K1 strain) parasites in vitro, and its moderate selectivity (against MRC-5 strain human cells) are summarized in Table 1. Natural compound **1** was originally isolated from a leaf of *Tabernaemontana dichotoma* in 1984 by the Verpoorte group [17] (Fig. 2). The relative structural determination of **1** was based on detailed NMR study, yet the absolute stereochemistry was not determined. As **1** has the potential to contain antimalarial activity, we decided to attempt the total synthesis of **1** to confirm its stereochemistry and investigate its antimalarial effect.

**Table 1:** Antimalarial activity and cytotoxicity of *Tabernaemontana dichotoma* extract

	IC_50_ (μg/mL)				
	Antimalarial activity		Cytotoxicity	Selectivity index (SI)	
	K1^a^	FCR3^b^	MRC-5	M/K^c^	M/F^d^
*Tabernaemontana dichotoma* MeOH extract	0.59	0.35	>25.0	>42.4	>71.4
Artemisinin	0.006	0.006	45.2	7528	7528

^a^Chloroquine-resistant strain

^b^Chloroquine-sensitive strain

^c^MRC-5/K1

^d^MRC-5/FCR3

In this review, the total synthesis, stereochemical determination, and antimalarial activity of 16-hydroxy-16,22-dihydroapparicine are discussed [18, 19].

**Fig. 3:**
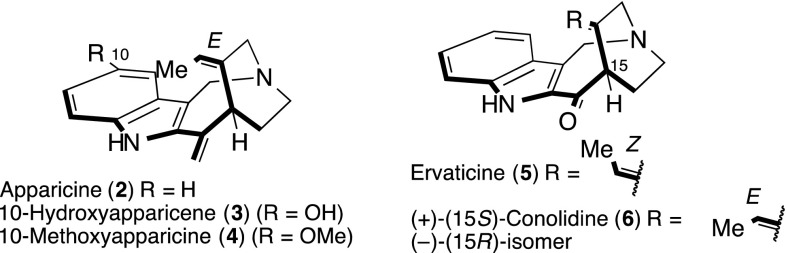
Structure of apparicine (**2**) and related compounds

Naturally occurring compound **1** has the same framework as Apparicine (**2**), the first 5-nor stemmadenine alkaloid discovered, which was isolated from *Aspidosperma dasycarpon* more than 45 years ago [20, 21] (Fig. 3). There are currently 22 known 5-nor stemmadenine alkaloid compounds [22–32], with the compounds exhibiting a wide range of biological activity, including being antimicrobial [33–35] and antibacterial (antituberculoid) [32], as well as displaying opioid properties [36]. Consequently, these alkaloids are of considerable interest. The main structural feature of the alkaloids is the strained 1-azabicyclo[4.2.2]decane skeleton, including a single carbon connection, between the indole 3-position and aliphatic nitrogen moiety, which is a defining characteristic of these compounds. The relative stereochemistry of **2**–**5** has also been reported for conolidine (**6**), the completed asymmetric total synthesis being accomplished by Micalizio’s group [37].

## Proposed biosynthesis

The special architecture involved, embodying a 1-azabicyclo[4.2.2]decane, is probably the result of the C-5 tryptamine atom being excised from the alkaloid stemmadenine by a retro-Mannich reaction. Some in vitro transformations of stemmadenine-type to 5-nor stemmadenine-type alkaloids have provided further support for this biogenetic model, which the following summarizes.

**Scheme 1:**
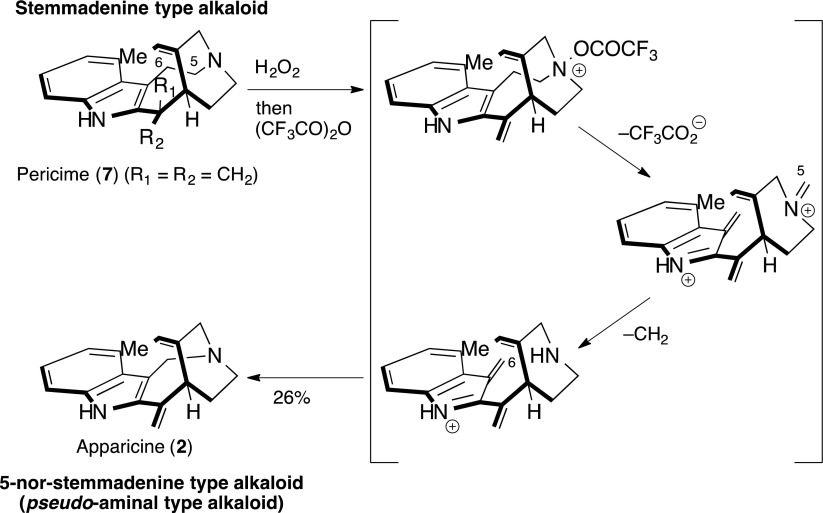
Biomimetic transformation

Kutney and co-workers reported the biosynthesis of the 1-azabicyclo[4.2.2]decane structure in the 5-nor stemmadenine alkaloids 50 years ago, using incorporated radioisotope experiments on the plant *Aspidosperma pyricollum*. Later, Lim and co-workers [38] reported partial synthesis of the *pseudo*-aminal type indole alkaloids, such as apparicine (**2**), using Potier’s expected biomimetic oxidative transformation from pericine (**7**) (Scheme 1).

## Synthesis studies

**Scheme 2:**
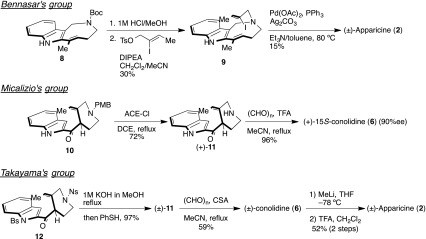
Reported total synthesis of apparicine and conolidine

**Scheme 3:**
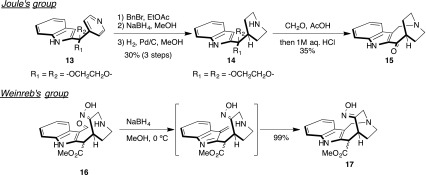
Reported synthetic study of the 1-azabicyclo[4.2.2]decane skeleton

Due to their unique structure and potentially useful biological activity, the total synthesis of 5-nor stemmadenine alkaloids has been reported by Bennasar et al. [39, 40], Micalizio [37], and Takayama [41] (Scheme 2). In addition, synthetic work on the 1-azabicyclo[4.2.2]decane skeleton core has been published by Joule [42, 43] and Weinreb’s group [44] (Scheme 3). A recent report of the total synthesis of (±)-apparicine (**2**) by Bennasar and co-workers [39, 40] detailed an approach which utilized an intramolecular Heck reaction. Micalizio and Takayama [37, 41] reported the total syntheses of conolidine (**6**), which could be derived from an iminium ion under intramolecular Mannich reaction. In addition, Micalizio and co-workers [37] clarified the absolute stereochemistry of **6**.

In 1977, Joule and co-workers [42, 43] reported the synthesis of apparicine, detailing an approach to **2** which utilizes an intramolecular Mannich cyclization to construct the 1-azabicyclo[4.2.2]decane skeleton. Weinreb’s group [44] reported the construction of a 4-cyclic compound **17** using nitrosoalkene and indole in 2014.

**Fig. 4:**
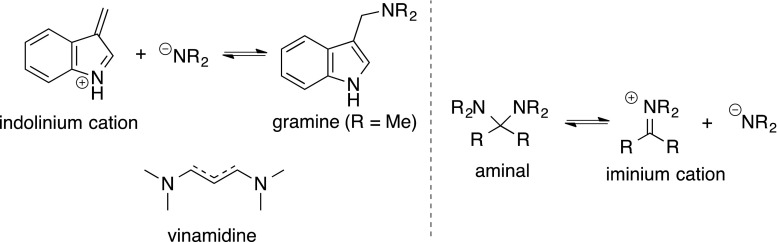
Gramines (vinamidines) as versatile *pseudo*-aminal type compounds

Our synthetic approach used a distinctive reaction based on the hypothesis that the main structural feature of these alkaloids is the strained 1-azabicyclo[4.2.2]decane skeleton, including a single carbon connection between the indole 3-position and aliphatic nitrogen moiety, which is a gramine-type (or vinamidine-type) moiety (Fig. 4). This structure has a “push–pull” nature, which is stabilized by electron-donating or electron-withdrawing groups. For example, the aliphatic carbon–nitrogen bond of the gramine type (or vinamidine type) is easily cleaved by retro-Mannich reaction under acid [45], base [46–48], and thermal [49] conditions, and with various reagents (e.g., trialkylphosphine [50–55], Lewis acid [56], phthalimide [57], thiol [58, 59], and activated ester [60, 61]) to generate the indolinium cation. We, therefore, anticipated that the propendiamine moiety was an indicator of reactivity similar to the aminal, leading us to suppose the framework as a “*pseudo*-aminal type structure”.

**Scheme 4:**
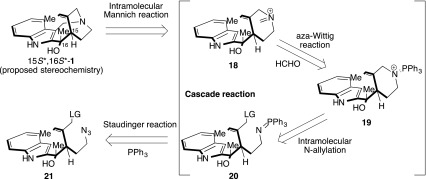
Designed novel phosphineimine-mediated cascade reaction

**Scheme 5:**
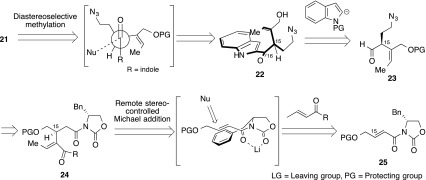
Retrosynthetic analysis of key intermediate

To complete the total synthesis of (15S*,16S*)-16-hydroxy-16,22-dihydroapparicine (**1**), we designed a novel phosphineimine-mediated cascade reaction, without any isolated unstable intermediate (Scheme 4). The cascade reaction sequence was: (1) Staudinger reaction of an azide **21** with triphenylphosphine to generate phosphineimine intermediate **20** [62]; (2) intramolecular N-allylation of phosphineimine transformed into aminophosphinium **19** [63–65]; (3) aza-Wittig reaction of **19** with formaldehyde; and (4) intramolecular Mannich reaction; nucleophilic attack might be performed from the indole 3-position to iminium cation **18**. We needed to solve two challenging issues. Firstly, the N-allylation of the phosphineimine group; phosphineimine has relatively high nucleophilicity, while the leaving group involves sufficient electrophilicity. Secondly, the formation of iminium cation using the aminophosphonium salt; there was no reported generation of iminium cation using the aminophosphonium salt and aldehyde via the aza-Wittig reaction. We found a solitary instance of the aminophosphonium salt with excess DMF to generating formamidinium salt [66]. However, the potential reactivity of the aminophosphonium salt has never been investigated. If we could overcome these challenges, an aminophosphonium salt (such as **19**) could become a useful reactant for the aza-Wittig reaction. The key precursor **21** could be prepared from diastereoselective methylation of 2-acylindole **22** with completion of the C-16 stereochemistry outcome of the Felkin–Anh transition state [67–71] (Scheme 5). Compound **22** could be constructed with the indole nucleophile and azidoaldehyde **23**.

To construct the C-15 stereocenter, we envisaged a remote stereocontrolled Michael reaction [72] of the α,β-unsaturated carboxamide **25** with the crotonic acid derivative.

**Scheme 6:**
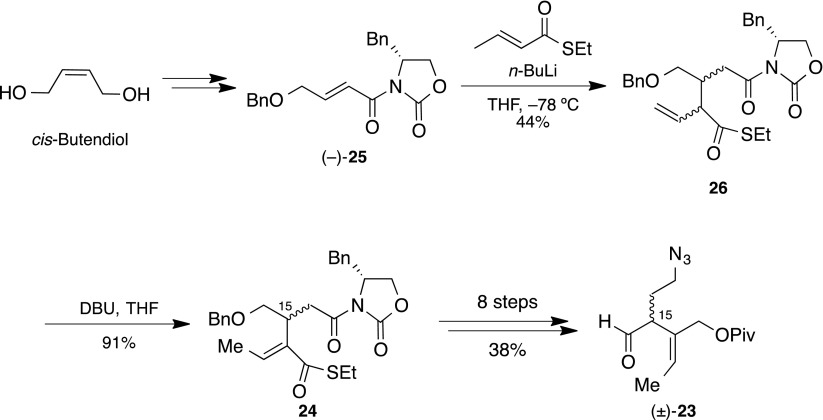
Synthesis of azidoaldehyde (**23**)

Synthesis of the azidoaldehyde **23** began from commercially available *cis*-butenediol, to afford (−)-**25** [73] (Scheme 6). With the Michael accepter in hand, we attempted the remote stereocontrolled Michael reaction of (−)-**25**, with only minor success, (−)-**25** appearing with no stereoselection and in low yield, along with γ-adduct as an undesired product. Subsequently, olefin isomerization afforded the unsaturated *E*-olefin **24** as a 1:1 diastereomixture (at C-15). Then, eight steps functionalization provided the azidoaldehyde (±)-**23** in 38 % overall yield.

**Scheme 7:**
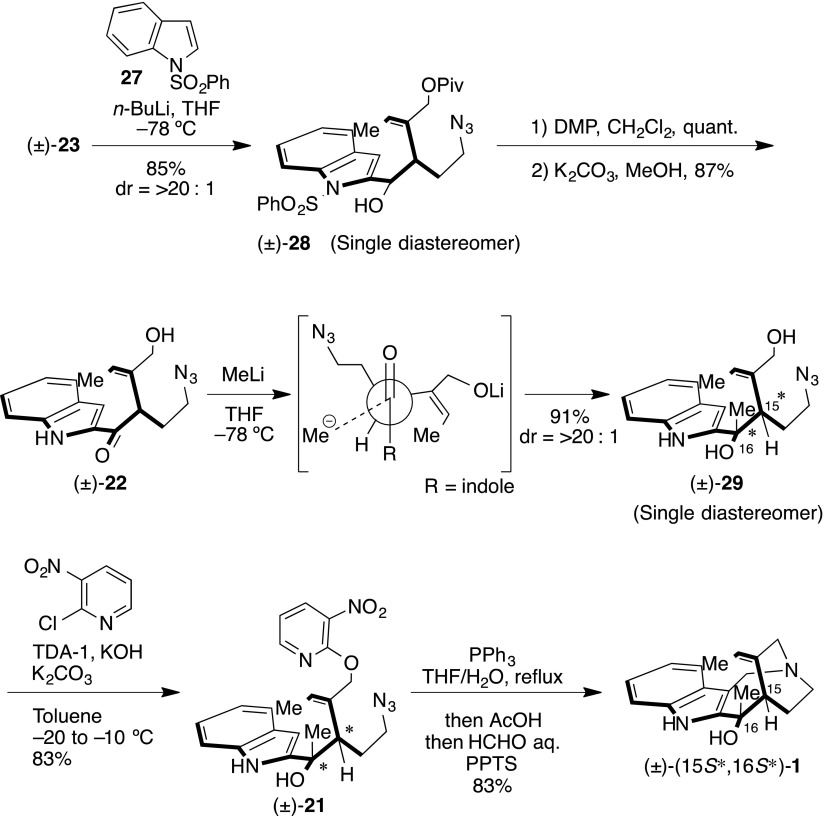
Synthesis of proposed hydroxyapparicine (**1**)

**Fig. 5:**
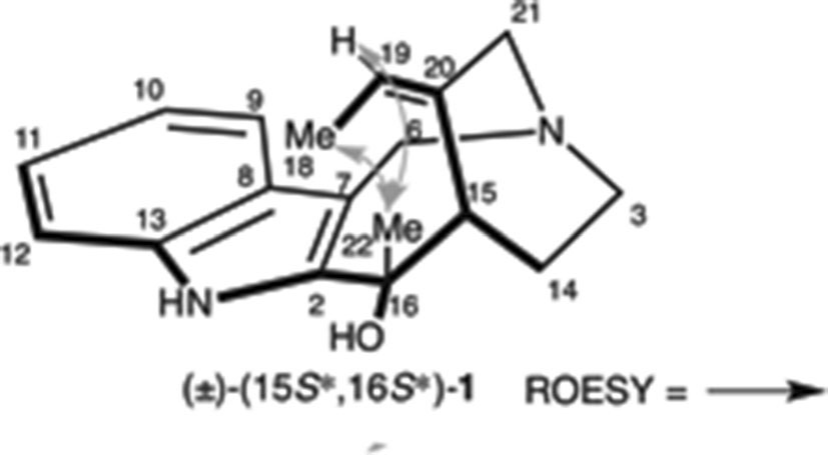
ROESY observations of synthetic (±)-(15S*,16S*)-**1**

With the azidoaldehyde (±)-**23** and the *N*-phenylsulfonyl indole **27** [74–81] in hand, we examined the nucleophilic addition, the hydroxyindole (±)-**28** being provided in 85 % yield as a single diastereomer (Scheme 7). Following the oxidation of (±)-**28** to obtain the (±)-ketoindole, the *N*-phenylsulfonyl and pivaloyl groups were subsequently removed under basic solvolysis to provide the hydroxyketoindole (±)-**22** in 87 % yield. Diastereoselective methylation of (±)-**22** converted it to dihydroxyindole (±)-**29** as a single diastereomer in excellent yield. The planar structure of (±)-**29** was confirmed by HMQC and HMBC studies. We expected the stereoselectivity outcome to be the Felkin–Anh transition state and so sought a suitable leaving group on the allyl alcohol. We eventually discovered a 3-nitropyridyl group [82, 83] as an efficient leaving group, allowing conversion of the 3-nitropyridinylation of (±)-**29** into the cascade reaction precursor (±)-**21** in 93 % yield, using the process reported by Ballesteros and co-workers [84, 85]. We then attempted construction of the 1-azabicyclo[4.2.2]decane skeleton, including the *pseudo*-aminal moiety. The cascade reaction precursor (±)-**21**, with PPh_3_ at 60 °C, generated iminophosphorane, the reaction mixture subsequently being acidified using AcOH for activation of the 3-nitropyridyl group. Finally, formaldehyde and PPTS were added to the reaction mixture to convert the iminophosphonium cation, followed by a Mannich reaction to furnish (±)-**1** in 88 % yield. The relative stereochemistry was confirmed by ROESY correlations (Fig. 5).

## Structure determination

However, the spectral data of synthetic (±)-**1** did not agree with that of naturally occurring **1** [17]. In particular, analysis of synthetic (±)-**1,** showed a ROESY relationship between H-18 or H-19 and 16-Me. Consequently, the relative stereochemistry of synthetic (±)-**1** was determined to be a 15S*,16S*-configuration. Data of synthetic (±)-(15S*,16S*)-**1** were then compared with naturally occurring compound (Table 2), with ^1^H and ^13^C NMR indicating differences of chemical shift (differences of all positions are shown in the experiment section). In ^1^H NMR, 16-Me and H-6α,β signals were registered more than 0.20 ppm and, furthermore, the ^13^C signals of the piperidine ring were greatly shifted from those seen in natural occurring **1**. Therefore, we expected that the 16-Me group in naturally occurring **1** was on the opposite face for the tri-substituted *exo*-cyclic olefin. Accordingly, the relative stereochemistry was anticipated to be the 15S*,16R*-configuration.

**Table 2:** Comparison of the NMR data of synthetic (±)-(15S*,16S*)-16-hydroxy-16,22-dihydroapparicine (**1**) with those reported for the natural product

Position	^1^HNMR			^13^CNMR		
	Synthetic (±)-(15S*,16S*)-**1** ^a^	Reported **1** ^b^	Δ*δ* ^c^	Synthetic (±)-(15S*,16S*)-**1** ^a^	Reported **1** ^b^	Δ*δ* ^c^
	*δ*_*H*_ (int., mult, *J* in Hz)	*δ*_*H*_ (int., mult, *J* in Hz)		*δ*_*C*_	*δ*_*C*_	
NH	8.30 (br s)	9.10 (br s)	−0.80	–	–	–
2	–	–	–	136.1	138.1	−2.0
3	3.04 (ddd, 14.0, 12.0, 7.0)	2.89–2.95 (m)	–	46.8	48.4	−1.6
	2.85 (dd, 14.0, 7.0)		–			
6	4.25 (d, 18.0)	3.95 (d, 17.5)	0.3	53.4	50.4	3
	4.58 (d, 18.0)	4.73 (d, 17.5)	−0.15			
7	–	–	–	109.4	107.3	2.1
8	–	–	–	127.9	129.9	−2.0
9	7.44 (d, 7.0)	7.46 (br d, 8.0)	−0.02	118.5	118.5	0
10	7.08 (ddd, 8.0, 7.0, 1.0)	7.18 (ddd, 8.0, 7.5, 1.0)	−0.10	119.2	119.2	0
11	7.20 (ddd, 8.0, 7.0, 1.0)	7.08 (ddd, 8.0, 7.5, 1.0)	0.12	122.6	122.3	−0.3
12	7.32 (ddd, 7.0, 2.0, 1.0)	7.33 (br d, 8.0)	−0.01	110.4	110.3	−0.1
13	–	–	–	135.3	135.2	0.1
14	1.87 (dddd, 14.0, 12.0, 7.0, 1.0)	2.01–2.22 (m)	-	25.0	23.4	1.6
	2.22 (dddd, 14.0, 11.0, 7.0, 2.0)					
15	3.35 (d, 7.0)	3.32 (dd, 3.5, 12.0)	0.02	44.0	43.2	0.8
16	–	–	–	76.2	74.5	1.7
18	1.75 (d, 8.6)	1.75 (ddd, 6.9, 2.5, 1.0)	0	13.7	13.8	−0.1
19	5.59 (br dq, 7.0, 1.0)	5.69 (q, 6.9)	–0.10	122.0	124.9	−2.9
20	–	–	–	136.1	134.5	1.6
21	3.79 (br d, 16.0)	3.66 (br d, 17.0)	0.13	55.1	53.2	1.9
	3.64 (br dq, 16.0, 2.0)	3.58 (br d, 17.0)	0.06			
22	1.56 (s)	1.73 (s)	–0.17	30.1	30.2	−0.1

^a^Measured in CDCl_3_ (^1^H: 500 MHz, ^13^C: 125 MHz)

^b^Measured in CDCl_3_ (^1^H: 300 MHz, ^13^C: 75 MHz)

^c^Δ*δ* (*δ*Syn − *δ*Nat)

**Scheme 8:**
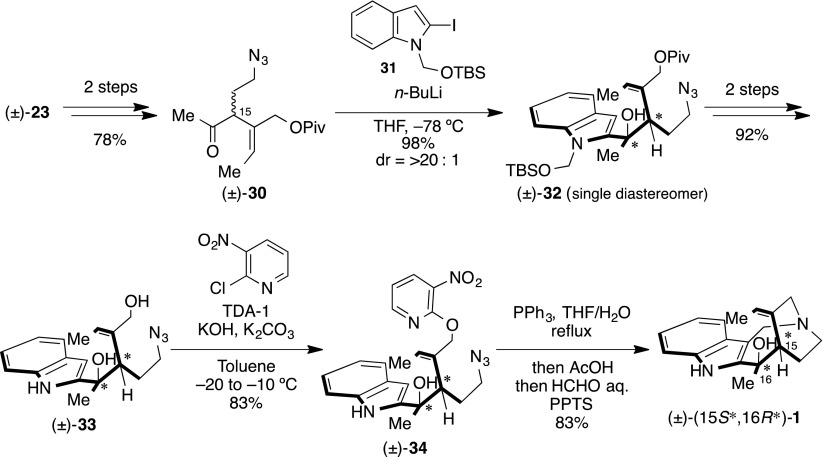
Total synthesis of (±)-(15S*,16R*)-**1**

**Fig. 6:**
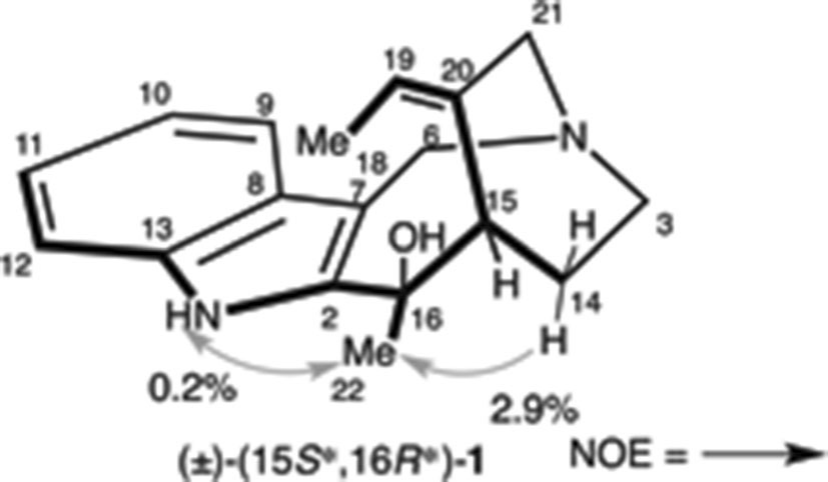
NOE observations of synthetic (+)-(15S*,16R*)-**1**

To confirm this consideration, we set about the synthesis of 15S*,16R*-isomer. The disputed stereocenter was prepared from ketoindole and methyl anion via the Felkin–Anh transition state. Therefore, the *R*-configuration could be constructed with methylketone (±)-**30** and indole nucleophile. We search and optimized nucleophilic addition using indole nucleophile. As a result, we found (*t*-butyldimethylsilyloxy)methyl (TBSOM) group [86–91] protected iodoindole as a suitable compound (Scheme 8). Hence, nucleophilic addition of **31** with (±)-**30** was converted into (±)-**32** in 97 % yield as a single diastereomer. The planar structure of (±)-**32** was confirmed by 2D NMR study. Subsequently, global deprotection of (±)-**32** obtained (±)-**33** in excellent yields. Following the same reaction sequence as the synthesis of (±)-(15S*,16S*)-**1** produced (±)-(15S*,16R*)-**1**. Characterization data provided for synthetic (±)-(15S*,16R*)-**1** were fully consistent with the data for the naturally occurring compound reported by Verpoorte and co-workers [17] (Table 3). In addition, an NOE relationship was observed between H-14a and H-22 (i.e., 16-Me) (Fig. 6).

**Table 3:** Comparison of the NMR data of synthetic (±)-(15S*,16R*)-16-hydroxy-16,22-dihydroapparicine; (±)-(15S*,16R*)-**1** with those reported for the natural product

Position	Synthetic (±)-(15S*,16R*)-**1** ^a^	Reported **1** ^b^	Δ*δ* ^c^	Synthetic (±)-(15S*,16R*)-**1** ^a^	Reported **1** ^b^	Δ*δ* ^c^
	*δ*_*H*_ (int., mult, *J* in Hz)	*δ*_*H*_ (int., mult, *J* in Hz)		*δ*_*C*_	*δ*_*C*_	
NH	8.42 (br s)	9.10 (br s)	−0.68	–	–	–
2	–	–	–	138.2	138.1	0.1
3	2.89–2.98 (m)	2.89–2.95 (m)	0	48.4	48.4	0
6	4.77 (d, 17.2)	4.73 (d, 17.5)	0.04	50.3	50.4	−0.1
	3.96 (d, 17.2)	3.95 (d, 17.5)	0.01			
7	–	–	–	106.9	107.3	−0.4
8	–	–	–	128.6	129.9	−1.3
9	7.47 (d, 8.0)	7.46 (br d, 8.0)	0.01	118.5	118.5	0
10	7.18 (dd, 7.5, 7.5)	7.18 (ddd, 8.0, 7.5, 1.0)	0	119.2	119.2	0
11	7.08 (dd, 7.5, 7.5)	7.08 (ddd, 8.0, 7.5, 1.0)	0	122.4	122.3	0.1
12	7.31 (d, 8.0)	7.33 (br d, 8.0)	−0.02	110.4	110.3	0.1
13	–	–	–	135.2	135.2	0
14	2.17 (m)	2.01–2.22 (m)	–	23.2	23.4	0.2
	2.02 (m)		–			
15	3.31 (dd, 3.2, 11.7)	3.32 (dd, 3.5, 12.0)	−0.01	43.1	43.2	−0.1
16	–	–	–	74.5	74.5	0
18	1.75 (d, 8.6)	1.75 (ddd, 6.9, 2.5, 1.0)	0	13.8	13.8	0
19	5.67 (q, 6.9)	5.69 (q, 6.9)	−0.02	125.2	124.9	0.3
20	–	–	–	134.1	134.5	−0.4
21	3.70 (d, 17.2)	3.66 (br d, 17.0)	0.04	53.1	53.2	0.1
	3.52 (d, 16.6)	3.58 (br d, 17.0)	−0.06			
22	1.74 (s)	1.73 (s)	0.01	30.1	30.2	−0.1

^a^Measured in CDCl_3_ (^1^H: 500 MHz, ^13^C: 125 MHz)

^b^Measured in CDCl_3_ (^1^H: 300 MHz, ^13^C: 75 MHz)

^c^Δ*δ* (*δ*Syn − *δ*Nat)

**Scheme 9:**
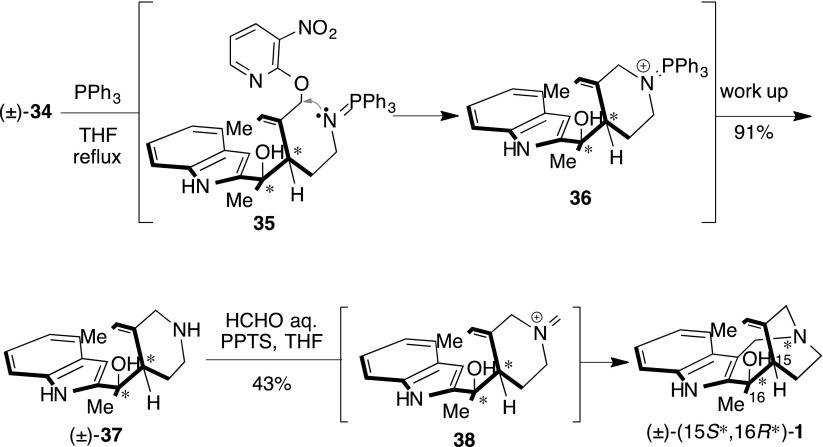
Stepwise synthesis of (±)-(15S*,16R*)-**1**

To clarify the cascade reaction mechanism, we attempted the experiment outlined in Scheme 9. At first, to provide the corresponding primary amine, a Staudinger reaction of (±)-**34** with PPh_3_ was carried out under reflux condition to obtain the piperidine-indole (±)-**37**, without acidic activation of the 3-nitropyridinyl group. ESI mass-monitoring of the first reaction allowed phosphineimine **35** to be easily generated from (±)-**34** and PPh_3_ without transformation into primary amine via solvolysis. In a time-dependent change, phosphineimine smoothly converted to the aminophosphonium cation **36**. Though the 3-nitropyridinyl group was a low electrophile, it was unnecessary for acidic activation. We inferred that the 1,3-allylic strain [92] was a key component, occurring via the tri-substituted olefin. Therefore, the 3-nitropyridinyl group was located within close proximity of the phosphineimine group. Subsequent intramolecular Mannich reaction of piperidine-indole (±)-**37** provided (±)-(15S*,16R*)-**1** in 43 % yield, using formaldehyde and PPTS. We subsequently expected that the aza-Wittig reaction of **36** with formaldehyde could assist in generating the iminium cation precursor **38** in a cascade reaction.

## Asymmetric total synthesis of 16-hydroxy-16,22-dihydroapparicine

**Scheme 10:**
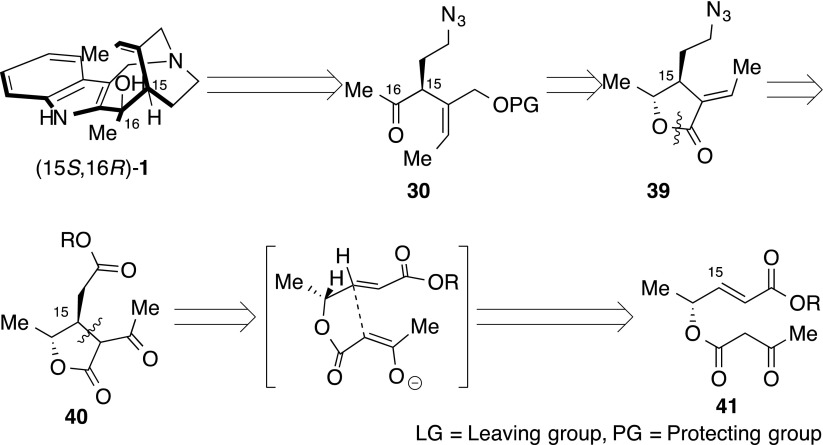
Asymmetric synthetic plan of (15*S*,16*R*)-**1**

We achieved the total synthesis of racemic 16-hydroxy-16,22-dihydroapparicine (**1**) and determined the true relative stereochemistry of the naturally occurring compound. In the next stage, we established the absolute stereochemistry of **1**. In order to accomplish asymmetric total synthesis, we used the chiral methylketone **30** (Scheme 10), which could be supplied from azidobutyrolactone **39**, including the appropriate functional groups. If **39** formed acetylbutyrolactone **40**, its acetyl and ester moiety could be transformed into *E*-ethylidene and azido groups, respectively. Acetylbutyrolactone **40** was, therefore, our key intermediate, with synthetic manners for related compounds having already been reported by Smith’s group and others [93–96]. We expected that **40** would involve a C-15 stereocenter being constructed by the intramolecular chirality transferring Michael reaction. We expected to perform via 5-*exo*-cyclization in the ketoester **41**, which should be stereo-specifically constructed by the Baldwin rule [97] and Thorpe–Ingold effect [98, 99].

**Scheme 11:**
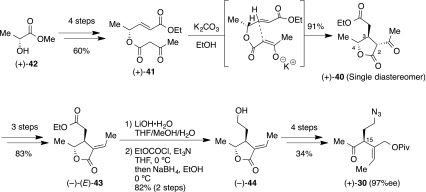
Asymmetric synthesis of methylketone (+)-**30**

Synthesis of the optically pure tri-substituted **40** began from commercially available (−)-(*R*)-methyl lactate **42**, which, after with four steps of preparation, provided the ketoester (+)-**41** in excellent yield (Scheme 11). With the optically pure (+)-**41** in hand, we attempted the intramolecular chirality transferring Michael reaction [100–104]. Through extensive optimization, we found a suitable condition to provide (+)-**40** in 91 % yield as a single diastereomer, and assignment of the relative stereochemistry was derived from the coupling constants and NOE correlation between α and γ protons. The key factor of the intramolecular chirality transferring Michael reaction was the solvent’s effect; polar solvent was stabilized to the anticipated transition state. The acetyl group of (+)-**40** converted into the ethylidene moiety along with the separable *Z*-isomer. The tri-substituted olefin moiety was determined to be of *E*-configuration by NOE correlation. The configuration of the C-3 stereocenter of (−)-**43** was determined after simple modification; hydrogenation of (−)-**43** obtained a single diastereomer, and the stereochemistry was confirmed to be *S*-configuration by NOE and ROESY correlation. Compound (−)-**43** was transformed into primary alcohol **44** by stepwise preparation; at first, selective hydrolysis of the ethyl ester group under basic condition generated carboxylic acid, followed by the corresponding acid anhydride. The furnished carboxylic anhydride was immediately reduced to the desired (−)-**44** in 82 % yield over the two steps [105, 106]. Subsequently, four steps functionalization provided the chiral methylketone (+)-**30** in excellent yield without racemization. The optical purity of the (+)-**30** (97 %ee) was confirmed by chiral HPLC analysis. The *R*-isomer of (−)-**30** was prepared in the same asymmetric synthetic manner from (−)-(*S*)-methyl lactate.

**Scheme 12:**
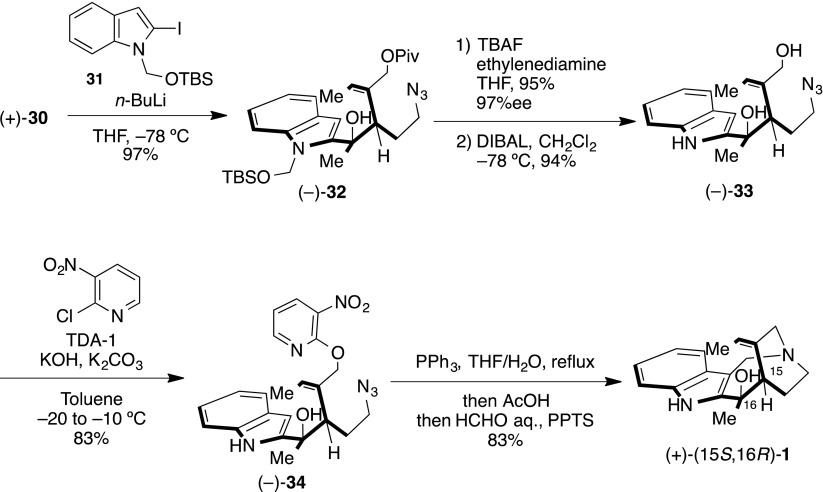
End game of the total synthesis of (+)-(15*S*, 16*R*)-**1**

Finally, (+)-**32** was exposed to the same procedure using (±)-(15S*,16R*)-16-hydroxy-16,22-dihydroapparicine **1** (Scheme 12). The cascade reaction precursor (−)-**36** underwent the same cascade reaction condition as that for the synthesis of (±)-(15S*,16S*)-**1**, (±)-(15S*,16R*)-**1** to give (+)-(15*S*,16*R*)-**1**. Characterization data proved that synthetic (+)-(15*S*,16*R*)-**1** was fully consistent with the data for the natural compound, as reported by Verpoorte and co-workers [17]. The optical rotation of synthetic (+)-(15*S*,16*R*)-**1**, [*α*]_D_^26^ +112.2 (*c* 0.9, EtOH), compared well with the values reported for the natural sample, [*α*]_D_^20^ +129 (*c* 0.1, EtOH), and the optical rotation of synthetic (−)-(15*R*,16*S*)-**1**, [*α*]_D_^26^ −104.2 (*c* 0.1, EtOH), was prepared in an asymmetric synthetic manner. In addition, an NOE relationship was observed between H-14a and H-22 (i.e., 16-Me). Therefore, the C-16 stereochemistry was determined to be the *R*-configuration.

## Biological activity

Naturally occurring and synthetic compounds were tested for antimalarial activity against *Plasmodium falciparum* parasites (chloroquine-resistant K1 strain and chloroquine-susceptible FCR3 strain) and for cytotoxicity (against human MCR-5 cells) [107–109], in comparison with the first-line antimalarial artemisinin.

The in vitro antimalarial activities and cytotoxicities of the naturally occurring and synthetic compounds are summarized in Table 1. As shown in Table 4, *Tabernaemontana* leaf extract (which includes (+)-(15*S*,16*R*)-16-hydroxy-16,22-dihydroapparicine) showed activity against both the chloroquine-resistant K1 strain and the chloroquine-sensitive FCR3 strains of *Plasmodium falciparum* (approximately 78-fold less potent than artemisinin, and with synthetic (±)-(15S*,16S*)-**1** having no measurable impact on chloroquine-susceptible parasites). Synthetic (±)-(15S*,16S*)-**1**, (+)-(15*S*,16*R*)-**1**, (−)-**1** displayed moderate to weak antimalarial activity (in the range of 9.0 to >12.5 μg/mL), while synthetic (−)-**1** and intermediaries showed minimal impact (7.17 to >12.5 μg/mL). The cytotoxicities against human cells of all synthetic compounds were weak (IC_50_ of 17–75 μg/mL), on average similar to that of artemisinin.

**Table 4:** Antimalarial activity of synthetic **1** and some intermediate compounds

	IC_50_ (μg/mL)				
	Antimalarial activity		Cytotoxicity	Selectivity index (SI)	
	K1^a^	FCR3^b^	MRC-5	M/K^c^	M/F^d^
*Tabernaemontana* leaf extract	0.59	0.35	>25.0	>42.4	>71.4
Synthetic (±)-(15S*,16S*)-**1**	>12.5	ND	33.3	>2.7	–
Synthetic (+)-(15*S*,16*R*)-**1**	9.00	8.37	51.2	5.7	6.1
Synthetic (−)-**1**	10.87	ND	75.2	6.9	–
(−)-(15*S*,16*R*)-**32**	>12.5	>12.5	ND	–	–
(−)-(15*S*,16*R*)-**39**	9.38	>12.5	54.0	5.8	<4.3
(−)-(15*S*,16*R*)-**33**	7.58	7.17	17.8	2.3	2.5
(−)-(15*S*,16*R*)-**34**	8.04	>12.5	>100.0	>12.4	8.0
(±)-(15*S*,16*R*)-**37**	8.98	>12.5	40.8	4.5	<3.3
Artemisinin	0.006	0.006	45.2	7528	7528

^a^Chloroquine-resistant strain

^b^Chloroquine-sensitive strain

^c^MRC-5/K1

^d^MRC-5/FCR3

The IC_50_ value of synthetic (+)-(15*S*,16*R*)-**1** proved to be significantly lower than the leaf extract containing naturally occurring (+)-(15*S*,16*R*)-16-hydroxy-16,22-dihydroapparicine.

## Conclusion

We achieved the first total synthesis of (+)-(15*S*,16*R*)-16-hydroxy-16,22-dihydroapparicine (**1**) and the (−)-enantiomer and determined the absolute stereochemistry of naturally occurring **1**. The synthesis involved a novel cascade reaction for efficient construction of the 1-azabicyclo[4.2.2]decane, including a *pseudo*-aminal moiety, via a Staudinger reaction, N-allylation, aza-Wittig reaction, and Mannich reaction. In addition, we developed a new method using diastereoselective 1,2-addition of methylketone, using *N*-TBSOM protecting the indole nucleophile and intramolecular chirality transferring Michael reaction with neighboring group participation. In particular, intramolecular chirality transferring Michael reaction proved to be a useful method for synthesis of the chiral tri-substituted butyrolactone. We established an effective enantioselective synthetic route for the production of *pseudo*-aminal alkaloids.

Synthetic (+)-(15*S*,16*R*)-**1** exhibited moderate/weak antimalarial activity against chloroquine-resistant *Plasmodium falciparum* parasites and there is a possibility that the structurally unique compounds may be useful for the development of novel antimalarial drug candidates.
